# The Double-Edged Sword of Anthropomorphism in LLMs ^†^

**DOI:** 10.3390/proceedings2025114004

**Published:** 2025-02-26

**Authors:** Madeline G. Reinecke, Fransisca Ting, Julian Savulescu, Ilina Singh

**Affiliations:** 1 Uehiro Oxford Institute, University of Oxford, Oxford OX1 1PT, UK; 2 Department of Psychiatry, University of Oxford, Oxford OX3 7JX, UK; 3 Department of Psychology, University of Toronto, Toronto, ON M5S 3G, Canada; 4 Department of Psychological and Brain Sciences, Boston University, Boston, MA 02215, USA; 5 Centre for Biomedical Ethics, Yong Loo Lin School of Medicine, National University of Singapore, Singapore 117597, Singapore

**Keywords:** hyperactive agency detection (HAAD), artificial intelligence, education, anthropomorphism

## Abstract

Humans may have evolved to be “hyperactive agency detectors”. Upon hearing a rustle in a pile of leaves, it would be safer to assume that an agent, like a lion, hides beneath (even if there may ultimately be nothing there). Can this evolutionary cognitive mechanism—and related mechanisms of anthropomorphism—explain some of people’s contemporary experience with using chatbots (e.g., ChatGPT, Gemini)? In this paper, we sketch how such mechanisms may engender the seemingly irresistible anthropomorphism of large language-based chatbots. We then explore the implications of this within the educational context. Specifically, we argue that people’s tendency to perceive a “mind in the machine” is a double-edged sword for educational progress: Though anthropomorphism can facilitate motivation and learning, it may also lead students to trust—and potentially over-trust—content generated by chatbots. To be sure, students do seem to recognize that LLM-generated content may, at times, be inaccurate. We argue, however, that the rise of anthropomorphism towards chatbots will only serve to further camouflage these inaccuracies. We close by considering how research can turn towards aiding students in becoming digitally literate—avoiding the pitfalls caused by perceiving agency and humanlike mental states in chatbots.

## 1 Introduction

In 1966, ELIZA became one of the world’s first chatbots—a computer program designed to convincingly mimic human conversation [1,2]. Though, technically speaking, ELIZA was a far-cry from the large language-based chatbots of today (e.g., Gemini, Chat-GPT), discussion surrounding these kinds of programs even then was remarkably prescient: “ELIZA shows, if nothing else, how easy it is to create and maintain the illusion of understanding, hence perhaps of judgment deserving credibility. A certain danger lurks there” [1]. In this paper, we address this danger, arguing that cognitive mechanisms related to agency detection and anthropomorphism make users of these technologies more likely to fall victim to such “illusions of understanding” within large language models (LLMs). We then consider why these tendencies may be pernicious when directed towards LLMs in an educational context. Though anthropomorphism may have some benefits on educational outcomes (e.g., retention; [3]), we argue that the rise of anthropomorphism in chatbots will only serve to further camouflage “hallucinations” and inaccuracies [4,5]. This makes anthropomorphism in LLMs serve as a “double-edged sword” for academic progress. In Section 4, we close by considering a potential intervention to mitigate people’s agentive and anthropomorphic tendencies towards LLMs.

## 2 Mechanisms of Anthropomorphism

### 2.1 Hyperactive Agency Detection

There is no shortage of cases where humans detect “agency” where there, in fact, isn’t any. An eroded hill on Mars gives the impression of looking like a face [6]; shapes following a random path give the impression of “chasing,” if oriented in a particular way [7]. Indeed, even the *suggestion* that letters or faces could be present in an image (that, in truth, consisted of pure noise) led participants to report seeing these illusory targets over a third of the time [8]. These kinds of effects are even present, to some extent, in human infants [9].

Why are humans so sensitive to agentive cues? One possibility is that, in the ancestral environment in which *Homo sapiens* evolved, it was adaptive to detect—and perhaps over-detect—agency [10,11]. Upon hearing a rustle in the grass, for example, it would be safest to assume that another agent (e.g., a lion) caused the noise. If this turned out to be false, then life would go on as normal. We would face no greater danger by having overestimated agency in this instance. But if we assumed the opposite, that the rustle came from the wind rather than a lion (when there was, in fact, a lion present), then we face grave risk. Though this evolutionary cognitive mechanism, termed “hyperactive agency detection” (HAAD), is most often discussed as a naturalistic explanation for religious belief [12–14], we see it as relevant also for understanding why people may be predisposed towards perceiving agency in LLMs.

Consider a study from Andersen et al. [15] examining hyperactive agency detection in the lab. Using virtual reality, researchers had participants traverse a forest with the aim of “detect[ing other] beings”. At the beginning of the study, researchers tricked participants into believing that there was a 5% or 95% chance of coming across another agent (when, in truth, there were never any other agents to be found). Researchers also manipulated the environment’s sensory reliability—changing whether the forest had clear conditions (high sensory reliability) or was clouded in mist (low sensory reliability). Though sensory reliability affected the false alarm rate—such that participants “detected” agents more often in the misty forest than the clear forest—what mattered most were their priors. Believing that there was a 95% chance of seeing another being in the forest, in fact, led participants to “see” such beings more often.

These findings deliver two primary insights for thinking about anthropomorphism in LLMs: First, there is a sense in which society currently stands in a technologically misty forest. There is widespread debate surrounding whether LLMs count as tools or as agents [16], whether they demonstrate “sparks of general intelligence” [17], and so on. Unlike a clear forest, where agents can be distinguished from non-agents with ease, people’s present experience with rapidly advancing artificial intelligence (AI) may mirror the misty forest, where much remains unclear and in flux. The second insight concerns the importance of people’s prior beliefs in detecting agency. When participants in this study *believed* that they had a high chance of encountering another agent, their experience in the forest aligned with these expectations. This may similarly occur in the context of LLMs. For example, former Google engineer Blake Lemoine became convinced that LaMDA, a large language model in development at the time, was sentient and deserved to be treated as a Google employee [18]. Lemoine’s convictions may have stemmed from how he interacted with LaMDA, such that his questions (e.g., “I’m generally assuming that you would like more people at Google to know that you’re sentient. Is that true?” [19]) confirmed his pre-existing beliefs about the model and its capacities.

Emerging research suggests that people do anthropomorphize ChatGPT [20], particularly with increased exposure [21]. People may even do so without explicit awareness [22]. This could lead to a concerning feedback loop, such that people’s anthropomorphic tendencies influence how they engage with LLMs (which then reinforces their beliefs about LLMs’ agentive qualities). This cycle, rooted in confirmation bias [23], would fuel the perception of agency where it likely does not exist—potentially affecting experts and non-experts alike [24,25].

### 2.2 Language as Agentive Cue

Large language models may invite anthropomorphism for another reason. Mimicking humanlike language and conversation is the cornerstone of what LLMs are designed to accomplish [26]. No longer is language use uniquely human [27]. This advance bolsters the likelihood of perceiving LLMs as agents: Communication, whether simple or complex, makes sense only within an agentive context [28]. Given that language likely evolved to allow agents to “share knowledge, thoughts, and feelings with one another” [29], language serves as arguably one of the most direct and perceptible reflections of others’ conscious minds. When we perceive others as capable of communicating in this way, the more likely we are to perceive them as agents with complex mental states [30].

Even preverbal infants make this link, inferring agency by observing whether a given entity can communicate. Across a swath of papers, having the ability to converse emerges as one of the most reliable means by which infants establish agency in others [31–35]. For example, in one study [31], experimenters presented 12–13-month-old infants with a live scene where an actor engaged in a contingent, back-and-forth interaction with a novel box covered in brown fur: The experimenter spoke to the box in human speech, and the box “spoke” back in fluctuating beeps, mimicking variability in natural language [36]. Despite the lack of morphological similarities to familiar agents, infants still treated the communicative box as an agent—following its “gaze” when it turned to the left or right. This kind of gaze following is common when infants interact with other people, but this behavior disappears when babies interact with inanimate objects [31–35]. Infants also rely more heavily on language use and conversational cues when determining agency as compared to other features—such as whether an entity has morphological similarities to other agents [37–39], the capacity to self-propel [40,41], or the capacity to engage in non-conversational contingent interaction with other agents [31]. This literature, taken together, suggests that humans may have an early-emerging, if not innate, tendency to link communicative capacity with agency. Given that contemporary LLMs far surpass this minimal conversational threshold, we anticipate that LLM output may very well trigger agentive inferences in users across the lifespan.

## 3 Anthropomorphism as a Double-Edged Sword in Education

Having a predisposition towards anthropomorphizing LLMs may be, on its own, value neutral. But this predisposition may open the door to a multitude of downstream harms for users (e.g., related to manipulation, coercion, and privacy [42]). We recognize that these harms may be especially rampant within an educational context, given students’ rapid adoption of these technologies as learning aids [43,44]. In the next section, we consider some of the consequences for education—both beneficial and detrimental—implicated in LLM-oriented anthropomorphism.

### 3.1 Benefits of Anthropomorphism

Though emerging research suggests that students’ use of ChatGPT can promote at least some aspects of engagement (e.g., completing tasks on time [45]), there has been little to no research examining the intersection between LLMs, anthropomorphism, and educational outcomes. In light of this gap, we draw on research regarding the effects of anthropomorphism in non-AI-based teaching materials. On the whole, this literature suggests that some amount of anthropomorphic detail in teaching materials may benefit students. In a series of studies examining the relationship between anthropomorphism in teaching illustrations and learning outcomes for primary and secondary school students, for example, Schneider et al. observed that having some amount (but not the maximum amount) of anthropomorphic content in illustrations improved retention and transfer learning. Anthropomorphism also enhanced students’ sense of intrinsic motivation [46]. In related work completed within secondary school and university settings, anthropomorphic teaching materials again outperformed non-anthropomorphic and control materials [3]. Though effect sizes vary across studies, a meta-analysis from Brom et al. examining the use of facial anthropomorphism and pleasant colors in teaching materials indicates that these inventions, on the whole, tend to improve academic outcomes (e.g., transfer, comprehension, retention), particularly for younger students [47]. These findings suggest that learners’ anthropomorphism of LLMs may be an asset for education, rather than a hindrance.

We note, however, that this literature suggests that even traditional learning materials can be too anthropomorphic. In research from Schneider et al., for example, students who received learning materials with the highest degree of anthropomorphism fared no better than students who received materials with the lowest degree of anthropomorphism. This may draw on students facing extraneous cognitive load in the high anthropomorphism case [46]. Further, in the above studies, all instances of anthropomorphism consisted of decorative additions to original teaching materials (e.g., static images of platelets with arms and legs [46]). Further research is needed to determine whether these benefits extend to LLMs in an educational context. At present, students in the United Kingdom most often use LLMs as “AI tutors”, engaging in back-and-forth conversation about course content [43]. This could, in principle, solve the famed “2 sigma” problem [48], where students with access to a personal tutor have been shown to outperform students taught in a traditional classroom setting by up to two standard deviations. Indeed, some researchers have already begun to implement and evaluate such LLM-based tutoring strategies, yielding promising results thus far [49,50]. Whether *anthropomorphism* in this context gives rise to beneficial educational outcomes, however, remains to be seen.

### 3.2 Drawbacks of Anthropomorphism

We also recognize that there is a range of educational disadvantages that may emerge from anthropomorphizing chatbots. We focus here on one chief concern: These systems remain prone to “hallucination,” meaning that they generate inaccurate statements that are endorsed by the model as correct [51]. Though this technical challenge may dissipate (or even disappear) with time, it continues to be an issue for the most cutting-edge models presently available. In a line of research testing how often LLMs would generate illusory citations (when specifically prompted to produce real and accurate citations), ChatGPT (powered by GPT-3.5 and GPT-4) hallucinated at least some of the time [5]. At its best, GPT-4’s reference list was 11% hallucinated; at its worst, its reference list was 43% hallucinated. GPT-3.5—the freely accessible model from OpenAI—fell even further short. Both of these models also struggled, to some degree, in applying “real citations [that were] appropriate for the answer to [the] question[s]” [5]. This is critical in an educational sphere: If a chatbot makes empirical claims with references, it should present the referenced material accurately.

Yet, GPT-4 provided “proper source[s] for answering [a given] question” only 57–89% of the time, depending on question specificity [5]. Insofar as hallucination remains a problem for LLMs, we see this as deep cause for concern.

This concern stems, in part, from the relationship between anthropomorphism and trust in artificial intelligence (e.g., [52–58]). When users of voice assistants, like Amazon’s Alexa, anthropomorphize their assistant (“[Alexa is] fun-loving”), they also tend to trust the assistant (“Although I may not know exactly how Alexa works, I know how to use it to perform tasks I want Alexa to do”) and treat their relationship with the assistant as more akin to a human relationship (“I feel guilty when asking too much of Alexa”; [57]). In a similar vein, people tend to trust autonomous vehicles more if the vehicles have been augmented with anthropomorphic features (e.g., having a voice, name, gender; [58]).

We expect these effects to translate to LLMs, such that people who see chatbots as more humanlike will likely trust both accurate and inaccurate AI-generated content more often. Indeed, evidence from Cohn et al. suggests that, after engaging with a pseudo-LLM, participants’ anthropomorphic beliefs predicted how trustworthy they found the LLM to be [52]. To be sure, students do report being aware that these systems can generate inaccurate content through hallucination [59]; there are even instances where student users would have done better academically if they had trusted LLM-generated content more [60]. But the problem remains: Students remain generally unaware of how often hallucination occurs [43], meaning that their trust in AI-generated content may be unfounded. We expect this challenge to become even greater as LLMs become further anthropomorphic [42], persuasive [61], and potentially even deceptive [62,63].

## 4 Reducing the Harm of Anthropomorphism

What can be done to mitigate the harms that students face as a result of anthropomorphizing LLMs, such as being more likely to trust hallucinated content? We speculate that promising interventions in other areas—such as those countering people’s acceptance of misinformation, more broadly—may also be successful here.

A growing amount of literature suggests that inoculating the public against misinformation by “preexposing them to severely weakened doses” of false content and then demonstrating their falsity [64]) can diminish susceptibility to misleading information [64–68]. For example, in a set of studies completed in the lab and in the field, researchers found that having people watch videos that deploy and then highlight common manipulation techniques—like using emotional language—actually reduced their willingness to share manipulative content on social media themselves [65]. This “inoculation” technique may dampen people’s vulnerability to misinformation across a variety of domains, including climate change [66,68] and vaccination attitudes [67]. Further, these benefits seem to persist beyond the initial intervention (e.g., [67]), suggesting that resistance to misinformation may last over time.

As applied to LLMs, an inoculation intervention might involve “prebunking” (i.e., providing a clear explanation of how LLMs work and, at times, hallucinate), followed by a “microdose” of misinformation from an LLM (that is then revealed to be hallucinated; [64]). For example, an instructor might show students an AI-generated analysis of *Hamlet*, which would likely, for the most part, be accurate. The instructor would then highlight any hallucinations that exist within the AI-generated text, such as an analysis of a scene that never existed. Demonstrating, through examples, how generative AI can convincingly produce true and false statements may help students build “attitudinal immunity” [69]. It may even be helpful to prebunk by describing humans’ natural inclination to anthropomorphize (and how this can contribute towards over-trusting AI systems), calling on the literature described in Sections 2 and 3. In research probing misinformation spread surrounding climate change, for instance, forewarning participants that “politically motivated groups use misleading tactics to try to convince the public that there is a lot of disagreement between scientists” was effective in strengthening their resistance to subsequent climate-related misinformation [66].

Though research is needed to determine whether an inoculation-based approach would be successful in this context, we expect that this kind of strategy may aid students in being digitally literate—by limiting their anthropomorphism through awareness of human psychology, and by having a healthy skepticism of AI-generated content. This approach dovetails with other calls to promote critical thinking and appropriate skepticism in Internet users, more broadly [70,71]. Given that individuals may vary in their propensity to anthropomorphize [72], as well as engage in critical thinking [73], we see this kind of intervention as valuable for investigation across users. Determining how to reduce people’s confidence in AI-based misinformation is an essential avenue for future work.

## 5 Conclusions

In sum, we see the rise of large language models as a remarkable technological achievement, one that comes with incredible promise and need for caution. In particular, we argue that certain cognitive mechanisms related to anthropomorphism—such as hyperactive agency detection and language as an agentive cue—may engender undue trust in chatbots. Further research should examine how users can be supported to calibrate their trust in LLMs, potentially through inoculation-based intervention. We see this as key for harnessing the benefits (and diminishing the drawbacks) of LLMs in the educational sphere and beyond.

## Data Availability

No new data were created or analyzed in this study. Data sharing is not applicable to this article.
